# A phase II study of personalized ultrafractionated stereotactic adaptive radiotherapy for palliative head and neck cancer treatment (PULS-Pal): a single-arm clinical trial protocol

**DOI:** 10.1186/s12885-024-13303-5

**Published:** 2024-12-21

**Authors:** P. Travis Courtney, Milisuryani L. Santoso, Ricky R. Savjani, Vishruth K. Reddy, Wanxing Chai-Ho, Maria A. Velez Velez, Deborah J. Wong, Christy Palodichuk, T. Vincent Basehart, Dylan P. O’Connell, Minsong Cao, Donatello Telesca, Robert K. Chin

**Affiliations:** 1 Department of Radiation Oncology, University of California, 200 Medical Plaza Driveway, Suite #B265, Los Angeles, CA 90025 USA; 2 Department of Medical Oncology, University of California, Los Angeles, USA; 3 Department of Biostatistics, Fielding School of Public Health, University of California, Los Angeles, USA

**Keywords:** Head and neck cancer, Stereotactic body radiotherapy (SBRT), Personalized Ultrafractionated stereotactic adaptive radiotherapy (PULSAR), Palliative radiotherapy

## Abstract

**Background:**

Many patients with head and neck cancer are not candidates for standard of care definitive treatments though often require palliative treatments given the frequent symptoms associated with head and neck cancer. While existing palliative radiotherapy regimens can provide adequate symptom control, they have limitations particularly with respect to local control which is becoming more important as advances in systemic therapy are improving survival. Personalized ultrafractionated stereotactic adaptive radiotherapy (PULSAR) is a novel radiotherapy regimen which leverages advances in radiotherapy treatment technology and extended interfraction intervals to enable adaptive radiotherapy and possible synergy with the immune system. Additionally, HyperArc© (Varian Medical Systems, Inc.) radiotherapy planning software allows for safe dose-escalation to head and neck tumors.

**Methods:**

This single-arm phase II study will prospectively evaluate PULSAR with HyperArc© software for palliative treatment of head and neck cancer. Patients with de novo or recurrent, localized or metastatic, head and neck cancer who are ineligible for or decline standard of care definitive treatments are eligible for enrollment. Forty-three patients will receive an 11 Gray fraction of radiation every two weeks for a total of five fractions and dose of 55 Gy. Adaptive radiotherapy planning is permitted. A safety and feasibility evaluation will be performed after enrollment of the first fifteen patients whereby the trial will be closed if five or more patients experience a CTCAEv5.0 grade 3 or 4 or any patient experiences a grade 5 toxicity probably attributable to PULSAR during or within three months after its completion. The primary endpoint is one-year local head and neck tumor control. Secondary endpoints include safety, disease progression-free and overall survival, symptomatic impact, frequency of re-simulation and/or adaptive planning, and radiation dosimetry of PULSAR. Additionally, enrolled patients are permitted to receive cancer-directed systemic therapy, including immunotherapy, during PULSAR which may allow for the analysis of the safety and efficacy of this combination.

**Discussion:**

The PULS-Pal trial is the first prospective study of PULSAR with HyperArc© software for head and neck cancer. We hypothesize that this radiotherapy regimen will lead to improved local tumor control compared with historical controls in patients undergoing palliative radiotherapy for head and neck cancer.

**Trial registration:**

Clinicaltrials.gov identifier: NCT06572423. Date of registration: August 28th, 2024.

**Supplementary Information:**

The online version contains supplementary material available at 10.1186/s12885-024-13303-5.

## Background

A significant proportion of patients with de novo or recurrent head and neck cancer (HNC) will not be candidates for standard of care definitive treatment(s) [1–3], which includes a combination of surgery, radiation therapy, and/or systemic therapy, and in some cases, standard of care stereotactic body radiotherapy (SBRT). Common contraindications for standard definitive treatment for HNC include locally advanced disease not amenable to definitive treatment because of the anticipated morbidity with primary or salvage treatment, poor performance status, presence of metastatic disease, or patient treatment preferences [4, 5]. In such cases, locoregional palliative radiation therapy is often utilized given the frequent accompanying symptoms of HNC such as pain or difficulty breathing or eating [6], with the most common HNC palliative radiation therapy regimens being the RTOG-8502 regimen (“quad-shot”) [7–9] and SBRT [10]. While these current palliative radiation therapy options can provide adequate symptom improvement, there are some limitations with these regimens as well.

With quad-shot, there are logistic challenges for both providers and patients given the twice-daily treatments, albeit for just two days total. Moreover, typically multiple cycles are needed to achieve maximal symptomatic benefit, although only about 50% of patients will be able to complete three or more cycles at which point the palliative response rate of this regimen is significantly increased [8, 11–13]. Additionally, while newer radiation therapy techniques such as intensity modulated radiation therapy (IMRT) have been employed to enhance the quad-shot regimen, it represents an older radiation therapy practice which may not fully leverage more recent advances in radiation therapy technology. Regarding SBRT, there is the possibility for severe toxicity, minimization of which is important in the palliative setting, as well as efficacy concerns, particularly for larger tumors or in the re-irradiation setting [6, 14–16]. However, a main limitation across all palliative radiation therapy regimens is suboptimal local control. Local tumor progression negatively impacts symptoms and possibly overall survival given the frequency at which locoregional tumor progression causes death in patients with HNC [17–19].

Prospective data of SBRT for unresectable head and neck cancer has demonstrated one year local control rates of 60% in the re-irradiation setting [20]. In the de novo setting, prospective data are limited and primarily consist of SBRT for early-stage glottis cancer, though in small, retrospective series predominantly for palliative purposes and/or for patients who are not candidates for standard of care definitive therapy, the reported one year local control rates range from 69–87% [21]. However, not all patients will be eligible for standard of care SBRT in the definitive or palliative setting, for example because of tumor size or location near the carotid artery [22]. In such cases, a variety of other radiation regimens are often used, such as quad-shot, which generally have lower and/or less durable local control rates [23–25]. The durability of symptom and local control in this patient population is becoming more important as advances in systemic therapy, particularly immunotherapy, are improving patient survival [26–29].

Personalized ultrafractionated stereotactic adaptive radiotherapy (PULSAR) is a novel radiation therapy regimen in which large radiation doses are delivered in “pulses” over extended interfraction intervals, with typically several days to multiple weeks between each radiation fraction [30, 31]. The PULSAR technique is enabled by advances in SBRT and image-guided radiation therapy, and allows for possible adaptation of each subsequent fraction to the most current patient anatomy and tumor response. This not only permits adequate time passage for tumor shrinkage and subsequent adaptive radiation therapy re-planning to occur, but also a protracted SBRT course may be less toxic than the standard SBRT course typically delivered over 1–2 weeks given the additional time for normal tissue healing as well as smaller radiation fields with possible tumor shrinkage. To this end, the PULSAR paradigm may enable safer tumor dose escalation [32]. Similarly, advances in radiation planning and treatment delivery, specifically HyperArc© (Varian Medical Systems, Inc.) [33] technology, have permitted dose escalation with high conformity in HNC SBRT [15, 34]. Dose escalation has been found to be associated with improved locoregional control in HNC [35, 36], and thus the combination of PULSAR with HyperArc© technology may further improve the therapeutic ratio of palliative radiation therapy in this patient population. Even more so, it has been suggested that there may be a synergism between PULSAR and immunotherapy, and further exploration of this relationship is warranted, particularly in the clinical setting [37]. With the increased use of immunotherapy in patients with recurrent, unresectable, or metastatic disease [38], the potential complimentary relationship between PULSAR and immunotherapy may provide these patients with additional benefit.

In this context, PULSAR is an appealing option for patients requiring palliative radiation therapy for HNC. Given the potential efficacy, safety, symptomatic, and logistical benefits of this radiation regimen, we hypothesize that PULSAR delivered with HyperArc© technology will result in improved local control and similar or improved symptom relief while mitigating toxicity risk compared with existing palliative radiation regimens in patients with de novo or recurrent HNC who are not candidates for standard of care definitive treatment(s), including standard of care SBRT. To test this hypothesis, we will conduct a prospective clinical trial of PULSAR in this patient population. Because PULSAR is a newer technique, it has not yet been comprehensively studied, particularly in combination with HyperArc© technology. To our knowledge, there are currently nine early phase clinical trials prospectively evaluating PULSAR in some capacity (NCT 04677413, 04779489, 04786093, 04889066, 05021237, 05846646, 05846659, 06044857, 06359275), none of which are in HNC. Thus, validation of its safety and feasibility is first required prior to larger-scale prospective studies of efficacy in HNC. Herein, we propose an early safety and feasibility study of palliative PULSAR delivered with HyperArc© technology in patients with de novo or recurrent, localized or metastatic HNC who are ineligible for or decline standard of care definitive treatment(s).

HyperArc© radiation technology is a commercial tool that provides automated non-coplanar volumetric modulated arc therapy (VMAT) treatment planning. It was initially developed for intracranial stereotactic radiosurgery; however, it can be adapted for the treatment of HNC, and our institution has evaluated HyperArc© technology in HNC dosimetrically and prospectively with encouraging results [15, 34]. From a treatment planning perspective, HyperArc© allows for target dose escalation, which as noted above is correlated with improved locoregional control, while maintaining minimal head and neck organs-at-risk doses compared with more conventional VMAT SBRT [34]. From a clinical perspective, our institution recently completed patient accrual for a phase II trial of HyperArc© SBRT to 55 Gray in five every other day fractions for definitive treatment in previously radiated patients with recurrent HNC (NCT03892720). The initial report of this study found this intervention to be feasible, safe, and well tolerated with minimal treatment-related toxicity and favorable quality of life metrics. Additionally, unpublished analysis of this study found a one year local control rate of 85%, which is higher than existing prospective data of SBRT for recurrent, previously radiated HNC reporting a one year local rate of 60% [20]. These findings support the rationale for using HyperArc© treatment planning technology in this study.

## Methods/design

### Study design

**Fig. 1:**
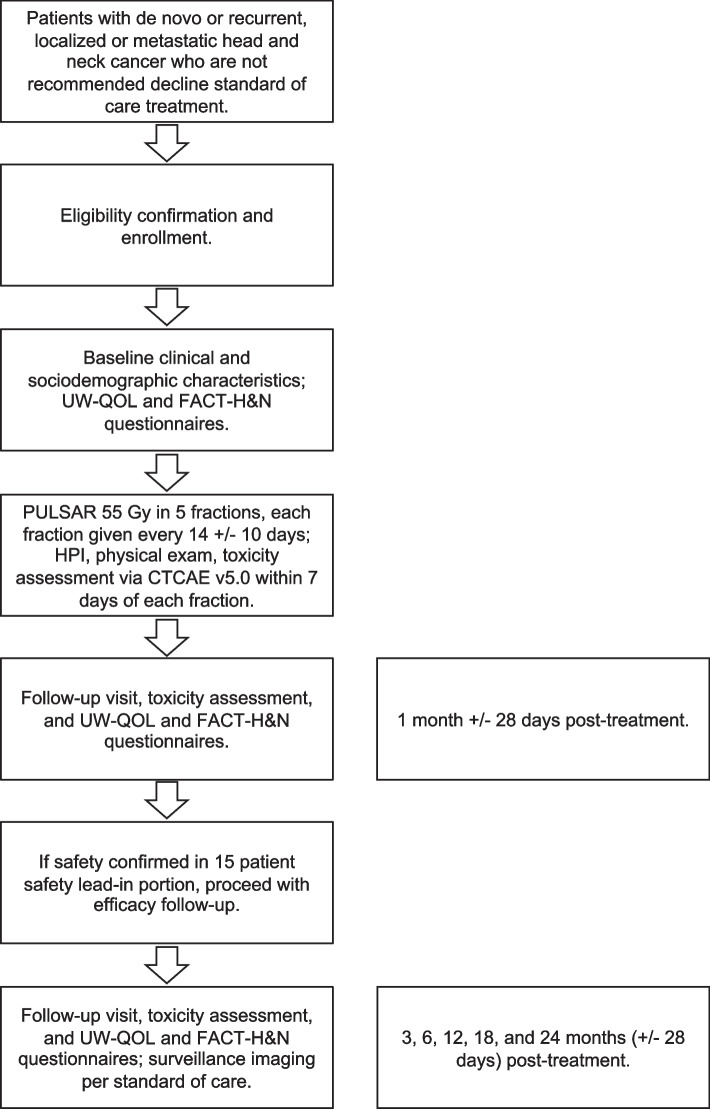
Trial Schema

This is a single-center (UCLA), single-arm, prospective phase II study with a safety lead-in. 43 subjects are planned. Each subject will receive the same PULSAR radiation therapy course as described below. The first 15 enrolled patients will be evaluated in the safety lead-in portion. If 5 or more of these 15 patients experience CTCAE v5.0 grade 3 or 4 or any patient experiences a grade 5 toxicity probably attributable to PULSAR during or within three months after completion of PULSAR, the study will be stopped for safety concerns; otherwise, the study will proceed, and those initial 15 patients will be included in the 43 subject sample. The trial schema is shown in Fig. 1.

### Ethics approval

This study is approved by the Institutional Review Board (IRB) of the University of California, Los Angeles (UCLA IRB #24–0663), and is registered at the U.S. National Institutes of Health (clinicaltrials.gov) # NCT06572423. The current protocol is version 1.6. This manuscript adheres to the guidelines and methodology outlined in the SPIRIT checklist.

### Aims

#### Primary aim

To evaluate the 1-year local tumor control of PULSAR for patients with de novo or recurrent, localized or metastatic head and neck cancer who are ineligible for or decline standard of care treatment(s), including standard of care SBRT.

#### Secondary aims

##### Secondary Aim 1

To evaluate the safety and toxicity of PULSAR as local treatment for patients with de novo or recurrent, localized or metastatic head and neck cancer who are ineligible for or decline standard of care definitive treatment(s).

##### Secondary aim 2

To evaluate the 1-year disease progression-free (PFS) and overall survival (OS) of patients with de novo or recurrent, localized or metastatic head and neck cancer who are ineligible for or decline standard of care treatment(s) who receive PULSAR.

##### Secondary aim 3

To evaluate the symptomatic impact of PULSAR as local treatment for patients with de novo or recurrent, localized or metastatic head and neck cancer who are ineligible for or decline standard of care definitive treatment(s).

##### Secondary aim 4

To determine the frequency of re-simulation and/or adaptive planning required with PULSAR.

##### Secondary aim 5

To evaluate standard of care tumor and organs-at-risk (OARs) dosimetry with PULSAR.

##### Secondary aim 6

To evaluate the safety and efficacy of combination PULSAR and cancer-directed systemic therapy in enrolled patients receiving cancer-directed systemic therapy.

### Patient selection

#### Study population

Patients with a diagnosis of primary or recurrent head and neck cancer who are ineligible for or decline standard of care definitive (i.e., curative) treatment(s), including standard of care stereotactic body radiation therapy (SBRT). Reasons for ineligibility for standard of care definitive treatments include locally advanced disease not amenable to standard definitive treatment(s) (e.g., due to the anticipated morbidity associated with definitive treatment), presence of metastatic disease, and medical comorbidities and/or poor performance status that preclude definitive treatment. Additionally, patients with primary or recurrent localized head and neck cancer who are candidates for but decline standard of care definitive treatment(s) will be eligible for enrollment.

#### Inclusion criteria

1.  ≥ 18 years old.
2. Diagnosis of primary or recurrent, localized or metastatic (AJCC 8th Edition stages I-IV) head and neck cancer. In primary diagnosis cases, pathologic confirmation is required. In recurrent and/or metastatic diagnosis cases, pathologic confirmation is not required if not beneficial to the patient as standard of care, and diagnosis can be assumed based on clinical and/or radiographic evidence.
3. Ineligible for or declines standard of care definitive treatment(s), which will be documented in the patient’s trial screening progress note in their electronic medical record by the treating physician.
4. Measurable disease within the head and/or neck clinically and/or on imaging studies (CT, PET, MRI) within 30 days from date of enrollment.
5. Patient maximum tumor(s) or tumor bed in postoperative patients, diameter must be less than 10 cm.
6. In a woman of childbearing potential, a negative serum or urine pregnancy test within 1 week of treatment start must be documented. Women of childbearing potential must agree to use adequate contraception (hormonal or barrier method of birth control; or abstinence) for duration of study participation and for up to 4 weeks following the study treatment.
7. Patients with a tracheostomy and/or a percutaneous endoscopic gastrostomy tube are eligible for inclusion.

#### Exclusion criteria

1. Pregnant or breast-feeding.
2. More than 1 prior radiation treatment course directed to the treatment area over the patient’s lifetime. In patients who have received 1 prior radiation treatment course directed to the treatment area, that prior radiation treatment course must have concluded at least 6 months prior to trial enrollment.
3. Any comorbidity or condition which would limit full compliance with the protocol.

### Registration and enrollment process

#### General guidelines

Patients with primary or de novo, localized or metastatic, HNC who are not eligible for or decline standard of care definitive treatment(s) will be informed of this clinical trial if eligible. The decision to participate will be voluntary. Eligible patients who decide not to participate will be offered alternative palliative radiation therapy regimens, systemic therapy (per their medical oncologist), alternative clinical trials, or palliative/hospice care. Information regarding this research study will be included on the UCLA Health Clinical Trials webpage and Clinicaltrials.gov.

#### Registration process

When feasible, an informed consent form in the patient’s preferred language will be given to the patient, or surrogate or legal-authorized representative when applicable, for review. Consent will be obtained after a clear and thorough discussion between the patient, or surrogate or legal-authorized representative when applicable, and the study investigator in clinic in the patient’s preferred language, utilizing a certified interpreter when necessary. To register a patient, the research coordinator will obtain or collect: (1) confirmation of diagnosis of HNC per inclusion criteria above; (2) signed informed consent form; (3) signed HIPAA authorization form. The informed consent form is provided in the Supplemental Material.

#### Pretreatment evaluations

Upon confirmation of eligibility and enrollment in the study, the following will be obtained prior to treatment (if not already performed):

- Medical history and clinical examination including weight and ECOG Performance Status
- Serum creatinine/estimated GFR within 60 days of CT simulation
- University of Washington Quality of Life (UW-QoL) and Functional Assessment of Cancer Therapy Head and Neck (FACT-H&N) validated questionnaire
- For patients who have received and/or are currently receiving cancer-directed treatment(s), evaluation for existing treatment-related toxicities as measured by the National Cancer Institute Common Terminology Criteria for Adverse Events Version 5.0 (CTCAE v5.0), as this version is currently implemented in our institution’s electronic medical record system which allows for easier and more consistent data documentation and recording.

Additional pretreatment studies including blood tests, imaging, and pathologic evaluation of tumor tissue for Human Papillomavirus and/or p16 positivity may be obtained per standard of care guidelines and/or the patient’s treatment team’s discretion.

### Intervention

#### Radiation simulation

After confirmation of eligibility, enrolled patients will undergo radiation CT simulation and planning per standard of care. IV contrast will be administered with CT simulation at the treating physician’s discretion though is not required. A thermoplastic face mask will be used for immobilization. HyperArc© technology will be used for treatment planning given prior internal validation of its superiority as above.

#### Radiation planning

For unresectable patients, the treating physician will delineate the gross tumor volume (GTV). For resected patients, a clinical target volume (CTV) will be delineated. The target will include the preoperative tumor volume with up to 5mm expansion for microscopic disease, with considerations for postoperative anatomical changes. Relevant previously obtained imaging studies may be fused to the CT simulation images as deemed necessary by the treating physician to further delineate the GTV or CTV. The GTV or CTV will be expanded by 3–5 mm for treatment setup error. This expansion will comprise the planning target volume (PTV). The dose will be prescribed to cover at least 95% of the PTV unless under-dosing of the PTV is required to meet constraints to OARs. Delineation of normal structures will be performed, including but not limited to: larynx, spinal cord, mandible, brainstem, skin, oral cavity, and parotid gland.

#### OAR dose constraints

No Prior Radiation Therapy to Tumor/Tumor Bed Site: will use max dose point constraints (defined as dose to 0.035 cm^3^) from the AAPM Task Group 101 Report [39].

Prior Radiation Therapy to Tumor/Tumor Bed Site (Reirradiation): will use the following max dose point constraints (defined as dose to 0.035 cm^3^) [15]:

1. Larynx: ≤ 20 Gy
2. Spinal cord: ≤ 8 Gy
3. Mandible: ≤ 30 Gy
4. Brainstem: ≤ 8 Gy
5. Skin: Dmax < 39.5 Gy

The treating physician has discretion when deciding between stated OAR constraints versus PTV coverage.

#### PULSAR treatment

Daily cone-beam CT will be obtained prior to radiation delivery to verify anatomic location and stability. Treatment will consist of 6, 10, or 15 MV photons directed at the PTV using HyperArc©’s automated non-coplanar VMAT technique. Patients will receive a total of 55 Gray in 5 fractions (biological effective dose with alpha/beta of 10: 115.5 Gray) to the PTV. Patients will receive each 11 Gray fraction of radiation every 14 +/−10 days. Treatment will be terminated early in cases of intolerable treatment-related toxicity, altered clinical context, or the patient declines further treatment.

#### Adaptive radiation planning

It is possible that patients will experience anatomical and/or tumor changes during the radiation therapy course such that patient alignment for radiation therapy is no longer optimized to the original CT simulation and radiation plan. In such cases where it is determined that adaptive planning is needed, we will abort planned radiation therapy and immediately perform re-simulation and offline re-planning if the anatomic change is significant enough to put adjacent normal tissue at significant risk; otherwise, we will re-plan offline with the next fraction of radiation to minimize treatment delays. Offline adaptive planning may include recontouring of target structures and/or OARs, and/or changes to the radiation prescription dose. This will be determined by the treating physician through standard of care clinical criteria and procedures. For patients who undergo re-simulation and re-planning, an extended window of 14 +21/−10 days from most recent radiation therapy fraction to receive the next radiation therapy fraction to accommodate re-simulation scheduling and re-planning time. The frequency of re-simulation and adaptive planning as well as associated changes in dosimetry will be measured and documented.

#### Cancer-directed systemic therapy

The following cancer-directed systemic therapy agents alone or in combination are permitted during and after PULSAR in the protocol:

1. Chemotherapy: 5-FU, capecitabine, carboplatin, cisplatin, cyclophosphamide, docetaxel, doxorubicin, etoposide, gemcitabine, hydroxyurea, methotrexate, paclitaxel, vincristine.
2. Targeted agents: Afatinib, cetuximab, dabrafenib erdafitinib, trametinib, vemurafenib.
3. Immunotherapy: Atezolizumab, cemiplimab, durvalumab, ipilimumab, nivolumab, pembrolizumab, tislelizumab, toripalimab.

The specific timing of administration of these cancer-directed agents with respect to delivery of PULSAR fractions, including the decision to hold the agent throughout the entirety of the PULSAR treatment, will be determined at the treating physician(s)’ discretion. Other systemic cancer-directed agents not listed above are permitted at the treating physician(s)’ discretion.

Daily oral systemic cancer-directed agents (e.g. BRAF and MEK inhibitors) may be taken during PULSAR at a frequency determined by the treating physician(s). Example recommendations are provided in the following reference: Guimond et al. [40]*, Adv Radiation Oncol*. However, coordination of stopping and starting daily oral medications around PULSAR fractions may be challenging, and holding such medications throughout the entirety of the PULSAR treatment is permitted at the treating physician(s)’ discretion.

#### Patient follow-up

**Fig. 2:**
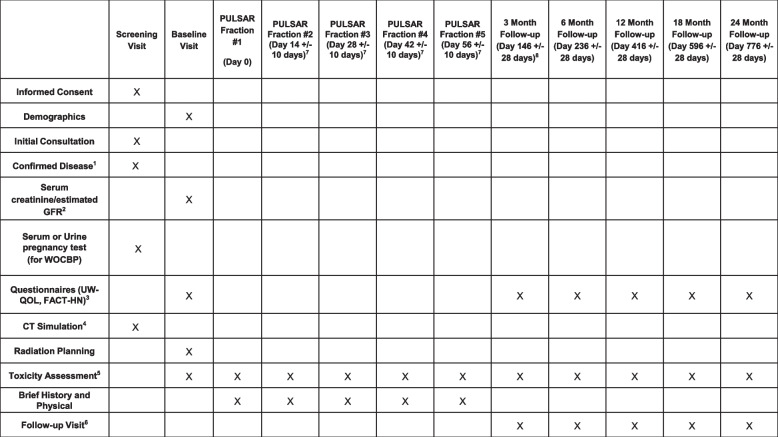
Trial Calendar. ^1^In primary diagnosis cases, pathologic confirmation is required. In recurrent and/or metastatic diagnosis cases, pathologic confirmation is not required if not beneficial to the patient as standard of care, and diagnosis can be assumed based on clinical and/or radiographic evidence. If available, Human Papillomavirus and/or p16 status will be collected and reviewed. ^2^Within 60 days of CT simulation. ^3^Questionnaires may be administered by telephone or electronically. ^4^May be repeated in between PULSAR fractions per treating physician’s discretion, as per section 6.5. ^5^During PULSAR course, toxicity assessments will occur within 7 days of each radiation fraction and may occur via telemedicine. ^6^May occur via telemedicine. ^7^For patients who undergo re-simulation and re-planning, an extended window of 14 +21/-10 days from most recent radiation therapy fraction to receive the next radiation therapy fraction to accommodate re-simulation scheduling and re-planning time. ^8^This will be the last follow-up for the safety lead-in portion, estimated to be at 12 months after trial opening. If proceeding to the phase II portion, the 15 patients in the safety lead-in portion will continue follow-up as per the study calendar, and all subsequently enrolled patients will follow the study calendar timeline and procedures. †Upon confirmation of eligibility and enrollment in the study, the following will be obtained prior to treatment (if not already performed)

Symptomatic response will be assessed through subjective patient report and completion of the UW-QoL and FACT-H&N questionnaires (Supplemental Material). Treatment-related toxicity during and after completion of PULSAR will be measured according to the CTCAE v5.0. Local tumor response will be assessed clinically and/or radiographically (e.g., by CT, PET, MRI), as applicable. Patient follow-up will be measured starting from the time of trial enrollment. Patient clinical assessments during PULSAR will occur within 7 days of each radiation treatment and consist of brief history, physical exam, and assessment of treatment-related toxicity. After receipt of the last PULSAR fraction, patients will be assessed clinically at 3 months +/- 28 days, 6 months +/- 28 days, 12 months +/- 28 days, 18 months +/- 28 days, and 24 months +/- 28 days post-treatment. These follow-up visits will consist of a history and physical exam, completion of the UW-QoL and FACT-H&N questionnaires, and measurement of toxicity. Surveillance imaging will be obtained per standard of care. Patients who experience disease progression will receive standard of care treatment(s) at the discretion of the treating physician. Patient enrollment, treatment, and follow-up is shown in Fig. 2.

### Adverse experience reporting and documentation

#### Adverse events

An adverse event (AE) is any untoward medical occurrence in a clinical investigation of a patient administered a pharmaceutical product and that does not necessarily have a causal relationship with the treatment. An AE is therefore any unfavorable and unintended sign (including an abnormal laboratory finding), symptom or disease temporally associated with the administration of an investigational product, whether or not related to that investigational product. An unexpected AE is one of a type not identified in nature, severity, or frequency in the current Investigator’s Brochure or of greater severity or frequency than expected based on the information in the Investigator’s Brochure.

The Investigator will probe, via discussion with the subject, for the occurrence of AEs during each subject visit and record the information in the site’s source documents. Adverse events will be recorded in the case report form (CRF). Adverse events will be described by duration (start and stop dates and times), severity, outcome, treatment and relation to study intervention, and the cause.

All adverse events should be treated appropriately. Such treatment may include changes in study intervention including possible interruption or discontinuation, starting or stopping concomitant treatments, changes in the frequency or nature of assessments, hospitalization, or any other medically required intervention. An assessment should be made at each visit (or more frequently, if necessary) of any changes in its severity, its suspected relationship to the study intervention, any of the interventions required to treat it, and its outcome.

#### AE evaluation

An investigator who is a qualified physician will evaluate all AEs. At each visit, the investigator will determine whether any AEs have occurred by evaluating the participant. Adverse events may be directly observed (through physical examination, laboratory test, or other assessments), reported spontaneously by the participant or by non-directive questioning of the participant at each study visit. The investigator must assess all AEs to determine:

- The severity grade according to CTCAE v5.0. If CTCAE grading does not exist for an adverse event, the severity of mild, moderate, severe, life-threatening and death (grades 1 - 5, respectively) will be used;
- Its relationship to the study intervention (related; probably-related; possibly-related; unlikely-related; not related);
- Its duration (start and end dates or if continuing at final exam);
- Action taken (none required; intervention not administered; dose reduced; dose delayed/interrupted; intervention discontinued);
- Treatment of adverse event (none required; medication given; procedure/surgery; other);
- Whether it constitutes a serious adverse event (SAE); and,
- Outcome (resolved; resolved with sequelae; persists, death; unknown).

The investigator’s assessment must be clearly documented in the study site’s source documentation with the investigator’s signature.

#### AE severity

The National Cancer Institute’s CTCAE v5.0 should be used to assess and grade AE severity, including laboratory abnormalities judged to be clinically significant. If the AE is not covered in the CTCAE, the guidelines shown in Table 1 below should be used to grade severity. It should be pointed out that the term “severe” is a measure of intensity and that a severe AE is not necessarily serious.

**Table 1:** AE severity grading

Severity (Toxicity Grade)	Description
Mild (1)	Transient or mild discomfort; no limitation in activity; no medical intervention or therapy required. The subject may be aware of the sign or symptom but tolerates it reasonably well
Moderate (2)	Mild to moderate limitation in activity, no or minimal medical intervention/therapy required
Severe (3)	Marked limitation in activity, medical intervention/therapy required, hospitalizations possible
Life-threatening (4)	The subject is at risk of death due to the adverse experience as it occurred. This does not refer to an experience that hypothetically might have caused death if it were more severe

#### AE relationship to the study intervention

The relationship of an AE to the study intervention should be assessed by an investigator using the following guidelines in Table 2. The causality assessment must be made based on the available information and can be updated as new information becomes available.

**Table 2:** AE relationship to study intervention

Relationship to intervention	Comment
Definitely	Previously known toxicity of the intervention; or an event that follows a reasonable temporal sequence from administration of the intervention; that follows a known or expected response pattern to the suspected intervention; that is confirmed by stopping or reducing the dosage of the intervention; and that is not explained by any other reasonable hypothesis
Probably	An event that follows a reasonable temporal sequence from administration of the intervention; that follows a known or expected response pattern to the suspected intervention; that is confirmed by stopping or reducing the dosage of the intervention; and that is unlikely to be explained by the known characteristics of the subject’s clinical state or by other interventions
Possibly	An event that follows a reasonable temporal sequence from administration of the intervention; that follows a known or expected response pattern to that suspected intervention; but that could readily have been produced by a number of other factors
Not Related/ Unlikely Related	An event that can be determined with certainty to have no relationship to the study intervention

#### AE action taken regarding study intervention

Where necessary the intervention name will be included in the source documenting the event.

- None required: No change in study intervention.
- Intervention not administered: The study intervention was held.
- Dose reduced: The study intervention was modified.
- Dose delayed/interrupted: The study intervention was temporarily stopped.
- Intervention discontinued: The study intervention was permanently stopped.

#### Treatment of the AE

- None: No treatment was required.
- Medication required: Prescription and/or over-the-counter medication was required to treat the AE.
- Procedure/Surgery: Medical procedure was required to treat the AE.
- Other: to be specified in the source documents.

#### AE outcome

- Resolved: The participant fully recovered from the AE with no residual effect observed.
- Resolved with Sequelae: The residual effects of the AE are still present and observable. Include sequelae/residual effects.
- Persists: The AE itself is still present and observable.
- Death: Death should be used when death is a direct outcome of the AE.
- Unknown: Information unavailable

#### Serious Adverse Event (SAE)

An SAE is any untoward medical occurrence that at any dose:

- Results in death,
- Is life-threatening, that is, places the participant at immediate risk of death from the event as it occurred. This definition does not include a reaction that, had it occurred in a more severe form, might have caused death;
- Requires inpatient hospitalization or prolongation of existing hospitalization, unless hospitalization is for:
  ◦ Routine treatment or monitoring of the studied indication, not associated with any deterioration in condition;
  ◦ Elective or pre-planned treatment for a pre-existing condition that is unrelated to the indication under study and has not worsened since signing the informed consent;
  ◦ Social reasons and respite care in the absence of any deterioration in the participant’s general condition;
- Results in persistent or significant disability/incapacity;
- Is a congenital anomaly/birth defect; or
- Is an important medical event.

Medical and scientific judgment should be exercised in deciding whether expedited reporting is appropriate in other situations, such as important medical events that may not be immediately life-threatening or result in death or hospitalization but may jeopardize the participant or may require intervention to prevent one of the other outcomes listed in the definition above. Examples include allergic bronchospasm, convulsions, and blood dyscrasias or development of drug dependency or drug abuse.

Note:

- Procedures are not AEs or SAEs, but the reason for the procedure may be an AE or SAE.
- Pre-planned (prior to signing the informed consent form [ICF]) procedures or treatments requiring hospitalizations for pre-existing conditions that do not worsen in severity are not SAEs.

For events that are serious due to hospitalization, the reason for hospitalization must be reported as the SAE (diagnosis or symptom requiring hospitalization). For deaths, the underlying or immediate cause of death should always be reported as an SAE. Any serious, untoward event that may occur subsequent to the reporting period that the investigator assesses as related to study intervention should also be reported and managed as an SAE. Unlike routine safety assessments, SAEs are monitored continuously and have special reporting requirements. All serious or intervention-related AEs ongoing at the end of treatment visit will be followed until resolution or until the condition is considered chronic/stable by the investigator.

#### AE and SAE reporting

All AEs and SAEs that occur between study enrollment and 6 months after completion of the intervention, will be reported in the eCRF and all SAEs will be reported in the Clinical Research Management System (CRMS) (OnCore). After 6 months after completion of the intervention, only AEs and SAEs suspected to be related to the intervention will be reported.

All events (serious and non-serious) must be reported with investigator’s assessment of the event’s seriousness, severity, and causality to the study intervention. A detailed narrative summarizing the course of the event, including its evaluation, treatment, and outcome should be provided. Specific or estimated dates of event onset, treatment, and resolution should be included when available. Medical history, concomitant medications, and laboratory data that are relevant to the event should also be summarized in the narrative. For fatal events, the narrative should state whether an autopsy was or will be performed, and include the results if available. Source documents (including medical reports) will be retained at the study site and should not be submitted to the Sponsor for SAE reporting purposes.

Disease progression/worsening of disease will not be recorded as an AE on the Adverse Event eCRF. However, events associated with disease progression may be recorded as AEs. Death due to disease progression should be recorded on the Death eCRF.

The study teams must report all SAEs to the JCCC DSMB in a timely manner. Regardless of relationship and expectedness of the event to the study intervention, the PI or their delegate must report the SAE within 10 business days of awareness. In the event of a participant death, the PI or their delegate must report the event within 2 business days of awareness. The SAE submission must note the date of awareness in the event narrative.

To report the SAEs to the JCCC DSMB, the study team enters the SAE information into OnCore, the CRMS. The CRMS generates and sends notifications regarding the submission to the JCCC DSMB administrative team and the study team. Study teams without access to OnCore, may complete the SAE submission form manually and submit it to the JCCC DSMB administrative staff, who will enter the SAE into OnCore on their behalf. The CRMS generates reports for full JCCC DSMB review.

The PI is also responsible for reporting any serious adverse event (SAE) to the sponsor, and any appropriate agency, according to the agency requirements. These include, but are not limited to: UCLA IRB; FDA; and NCI, if it is an NCI sponsored trial.

The collection period for all SAEs will begin after informed consent is obtained and end after procedures for the final study visit have been completed. Any SAEs experienced after this period should only be reported to the Sponsor if the investigator suspects a causal relationship to the study intervention.

In accordance with the standard operating procedures and policies of the local Institutional Review Board (IRB)/Independent Ethics Committee (IEC), the site investigator will report SAEs to the IRB/IEC when applicable.

#### DSMB review of AEs and SAEs

The JCCC DSMB reviews all AEs as part of the summary report review performed quarterly, semi-annually or annually based on the risk level assigned to the study and in accordance with the JCCC Data and Safety Monitoring Plan. The DSMB reviews each SAE monthly and determines if the event(s) warrants modifications to the protocol to ensure subject safety. In their review, the DMSB considers prior occurrences of similar toxicity with the intervention under study, as well as the severity of the event and the likelihood of relation to the study intervention or investigational product. The DSMB may recommend no changes to the study if the event is expected or related to other causes such as underlying disease. To assist discussions and decisions, the DSMB may request the expert advice of another clinical researcher with national experience.

### Data safety, collection, retention, and monitoring

#### Monitoring

The JCCC Office of Regulatory Compliance (ORC) will perform monitoring and auditing activities in accordance with the JCCC Data and Safety Monitoring Plan (DSMP).

#### Data safety monitoring

The JCCC DSMB will serve as the DSMB to review data relating to safety and efficacy, to conduct and review interim analyses, and to ensure the continued scientific validity and merit of the study, according to the JCCC DSMP. There will be quarterly, semi-annually or annual interim review(s) conducted by the DSMB based on the risk level assigned to the study for the purpose of monitoring study conduct and assessing participant safety. Risk level 1 studies are the highest risk and are reviewed quarterly; Risk Level 2 studies are intermediate risk and are reviewed semi-annually; Risk Level 3 studies are the lowest risk and are reviewed annually. Further details regarding the timing and content of the interim reviews via summary reports are included in the JCCC DSMP.

#### Data management

The radiation oncology research staff will be responsible for the database records of study patients. The data will be kept on the research staff computer, under password protection, with the patient information de-identified (study patients will be referred by their coded study number). A chart with all the relevant research patient information will be maintained for each patient by the research coordinator and will be filed in a locked cabinet. Only the research team (study staff, investigators, and project supporting staff) will have the password and key to the data from the study patients.

#### Confidentiality

Study data will be maintained in password protected computer files. Only research personnel will have access to this information. All identifiers will be removed. The patient’s name or other public identifiers will not be included in any information shared with other investigators. The master key that will identify specific study patients to their coded study number will be kept in a separate password protected file on the research staff computer. Only the study staff and the principal investigator will know the password to this file.

### Statistical methods and considerations

#### General

All analyses will be descriptive and will be presented by treatment period and overall as appropriate. Data collected in this study will be presented using summary tables and patient data listings. Continuous variables will be summarized using descriptive statistics, specifically the mean, median, standard deviation, minimum, and maximum. Categorical variables will be summarized by frequencies and percentages.

#### Safety lead-in/interim analysis

The study includes a safety lead-in portion, with formal evaluation on enrollment of the 15^th^ subject. The trial will be stopped if 5 or more patients in the safety lead-in portion experience a serious adverse event, defined as a CTCAE v5.0 grade 3 or 4 toxicity, or if any patient experiences a grade 5 toxicity probably attributable to the intervention during or within three months after completion of PULSAR.

#### Sample size determination

Prospective data of SBRT for unresectable head and neck cancer has demonstrated 1 year local control rates of 60% in the re-irradiation setting [20]. In the de novo setting, prospective data are limited and primarily consist of SBRT for early-stage glottis cancer, though in small, retrospective series predominantly for palliative purposes and/or for patients who are not candidates for standard of care definitive therapy, the reported 1 year local control rates range from 69–87% [21]. However, in the palliative setting or patients unfit for definitive therapy, a variety of other radiation regimens are often used, such as the “Quad-Shot” regimen, which generally have lower and/or less durable local control rates [23–25]. From these data, we estimate the historic control group 1 year local control rate to be 65%.

For the study group, unpublished analysis of a recently closed phase II trial at UCLA of dose-escalated SBRT to 55 Gray for treatment of previously radiated, recurrent head and neck cancer demonstrated a 1 year local control rate of 85% (NCT 03892720). We therefore hypothesize that patients enrolled in this trial will have a similar 1 year local control rate. A sample size of 38 patients will achieve 80% power to detect a 20% difference in the 1 year local control rate between PULSAR (85%) and historic control (65%), using a two-sided one-sample exact binomial test, at 0.05 significance level. Considering that some patients may become ineligible/drop out after enrollment, the target sample size is determined to be at least 43 patients. The 15 patients in the safety lead-in portion will be included in the sample should the study proceed past this portion.

#### Patient accrual and study duration

We used an institutional database of patients with HNC and departmental treatment records to retrospectively approximate the number of patients that would have been eligible for this trial. Taking into account errors in electronic medical record documentation and coding [41, 42], we estimate that approximately 120–170 eligible patients were seen in our department over the past five years, roughly equating to two to three eligible patients monthly. These numbers are in line with or even slightly lower than what we have seen clinically recently, particularly as the COVID pandemic has subsided and more patients are being seen in our department. Additionally, our department’s recently closed phase II study of definitive HyperArc© SBRT in patients with recurrent, previously radiated HNC enrolled and treated the first fifteen patients in one year [15]. The patient population in this recently closed phase II trial is much narrower than that in the proposed study, so we anticipate similar if not faster enrollment with this proposed study compared with the recently closed phase II trial. As such, we anticipate enrollment of two to four patients monthly and that it will take approximately 15 months to complete the safety lead-in portion and 4 years to complete the study. Study-related data will be stored after completion of the clinical portion of the study to be used in the exploratory portion of the study, as needed.

#### Populations for analyses

##### Full Analysis Set (FAS)

All efficacy analyses will be performed on the Full Analysis Set (FAS) following the Intent-To-Treat (ITT) principle. Patients who were enrolled but did not receive study treatment are included in the FAS population.

##### Evaluable Analysis Set (EAS)

A subset of the full analysis set (FAS), the evaluable analysis set is defined as those treated patients who are evaluable (i.e. compliant with treatment and without any major protocol deviations that may impact primary efficacy analysis). The EAS will be used for supportive analyses of the efficacy endpoints.

##### Safety Analysis Set (SAS)

All patients who received at least 1 fraction of radiation and for whom any valid post-baseline safety data are available will be included in the safety analysis set. When assessing safety and tolerability, summaries will be produced based on the safety analysis set. Patients are analyzed according to the actual treatment received.

#### Study endpoints

##### Primary endpoint

The primary endpoint is local control rate of the treated tumor target at 1 year, as measured from the date of enrollment. Local control will be evaluated by radiographic and/or clinical assessment as applicable. Participants who experience death or are lost to follow-up prior to 1 year will be assessed by their last radiographic/clinical evaluation.

##### Secondary endpoints

1. Measurement of grade 3 or higher treatment-related toxicity during or within 24 months after completion of PULSAR, according to the CTCAE v5.0.
2. Disease progression free survival (PFS), defined as the time from enrollment to the first objectively documented disease progression, either in or out of the treated field per radiographic and/or clinical evaluation, or death due to any cause. Participants who experience death or are lost to follow-up prior to 1 year will be assessed by their last radiographic/clinical evaluation.
3. Overall survival (OS), defined as the time between enrollment and death of any cause. Participants who are lost to follow-up prior to 1 year will be censored at the date of their last documentation in the medical record system.
4. Impact of PULSAR on symptoms/quality of life as measured by the UW-QoL and FACT-H&N validated questionnaires.
5. Measurement of the frequency of re-simulation and/or adaptive planning required with PULSAR.
6. Evaluation of the standard of care tumor and OAR dosimetry with PULSAR.
7. Evaluation of the safety and efficacy of combination PULSAR and cancer-directed systemic therapy in enrolled patients receiving cancer-directed systemic therapy will be descriptive and exploratory.

#### Statistical analysis plan

##### Demographics and baseline characteristics

Demographic data, medical history, other baseline characteristics, and concomitant disease will be summarized. To determine whether the criteria for study conduct are met, corresponding tables and listings will be provided. These will include an assessment of protocol deviations, study treatment accountability, and other data that may impact the general conduct of the study. The analysis sets FAS, EAS, SAS will be used.

##### Efficacy analyses

1. Primary endpoint: Primary endpoint analyses will be performed on patients included in analysis sets FAS and EAS across the whole cohort, as well as separate analyses stratifying patients by Human Papillomavirus/p16 status and receipt of cancer-directed systemic therapy, both in general and by agent type (chemotherapy, targeted, immunotherapy). For the primary endpoint, we will calculate the percentage and construct 95% exact confidence interval for local control rate. Additionally, Kaplan–Meier method and cumulative incidence functions considering distant progression or death as a competing risk will be used to provide estimates of the local control at 1-year, median local control, and cumulative incidence of local control at 1-year, with corresponding 95% confidence intervals.
2. Secondary Endpoints: Secondary endpoint analyses will be performed on patients included in analysis sets FAS and EAS across the whole cohort, as well as separate analyses stratifying patients by Human Papillomavirus/p16 status. For the secondary endpoints of PFS and OS, Kaplan–Meier method and cumulative incidence curves treating death as a competing risk will be used to provide estimates of the PFS/OS at 1-year, median PFS/OS, and cumulative incidence of PFS/OS at 1-year. Corresponding 95% confidence intervals will also be presented.

#### Safety analyses

Safety analyses will be performed for patients included in analysis set SAS. Percentage of patients with acute or chronic grade 3 or higher treatment-related toxicity, incidence of AEs, clinical laboratory information, vital signs, ECOG performance status, and weight, will be tabulated and summarized. Incidence of AEs will be summarized overall and with separate summaries for SAEs, AEs leading to discontinuation, AEs leading to death, etc. The overall safety and tolerability will be assessed throughout the study period. All AE data will be listed individually by patient identifier.

#### Symptom/quality of life analyses

Analysis of data related to the symptom/quality of life impact of PULSAR using the UW-QoL and FACT-H&N validated questionnaires will be primarily descriptive and exploratory.

#### Descriptive/exploratory analyses

Analysis of data related to the frequency of re-simulation and/or adaptive planning required with PULSAR; standard of care tumor and OAR dosimetry with PULSAR; and the safety and efficacy of combination PULSAR and cancer-directed systemic therapy in enrolled patients receiving cancer-directed systemic therapy will be descriptive and exploratory.

### Dissemination policies

#### Protocol modifications

Important protocol modifications will be communicated to relevant parties including investigators, the UCLA IRB, trial participants, journal, and regulators by standard methods of communications.

#### Trial results

The investigators will communicate trial results to participants, healthcare professionals, the public, and other relevant groups via publication.

#### Data

Public access to the full protocol and statistical code will be granted.

## Discussion

A key component of the PULSAR technique is the ability for radiotherapy plan adaptation, which may not be fully leveraged in this trial. While this protocol permits offline adaptive radiotherapy planning, it does not include online adaptive radiotherapy planning as the technology for online adaptation is not available on the linear accelerator (TrueBeam STx, Varian Medical Systems, Inc.) on which HyperArc© software is compatible that will be used in this single-institution trial. Furthermore, there are other advantages with PULSAR which may supplant its safety, efficacy, and feasibility. PULSAR offers a relatively flexible and convenient schedule for patients, which is particularly important for patients receiving palliative treatments for whom time outside of medical visits may be prioritized, and adding additional visits for the re-simulations required for more frequent offline adaptive planning may diminish this benefit and hinder enrollment. Additionally, HyperArc© treatment planning software provides steep radiation dose falloff, and aggressive adaptive re-planning for each PULSAR fraction could increase the risk of local failures by missing microscopic disease. Furthermore, while there may be a benefit to more frequent adaptation with this protocol, it is currently unclear how meaningful this benefit is in the head and neck cancer setting in which OARs are relatively immobile compared with other sites such as pelvic malignancies. Another benefit of PULSAR is its possible immune system synergy, which is unrelated to radiotherapy adaptation. Future studies and iterations of this intervention may seek to incorporate more frequent, ideally online, adaptive radiotherapy.

In conclusion, this study has significance across a wide-range of clinical situations. In regards to the enrolled patient population, there is a need for more optimal palliative treatments, especially as longevity is improved from advances in other treatment modalities, particularly systemic therapy. As such, if the safety and efficacy of PULSAR is confirmed, it may supplant existing palliative radiation therapy regimens for HNC and become more common in clinical practice. Furthermore, there are limited prospective clinical data evaluating the PULSAR regimen, and this study will provide guidance on the logistics, workflow, safety, and efficacy of PULSAR with HyperArc© technology so that it may be studied and utilized further in HNC and/or in other cancer sites. Similarly, given the interest in the combination of PULSAR and immunotherapy, this study could provide early, hypothesis generating data on this combination treatment, laying the groundwork for future studies. Ultimately, we anticipate that this study will provide important, early data on this promising new radiotherapy paradigm.

## Supplementary Information

Supplementary Material 1.

## Data Availability

No datasets were generated or analysed during the current study.

## Abbreviations

AAPM: American Association of Physicists in Medicine
AE: Adverse Event
AJCC: American Joint Committee on Cancer
CRF: Case Report Form
CRMS: Clinical Research Management System
CT: Computed Tomography
CTCAE: Common Terminology Criteria for Adverse Events
CTV: Clinical Target Volume
DSMB: Data Safety and Monitoring Board
DSMP: Data Safety and Monitoring Plan
EAS: Evaluable Analysis Set
ECOG: Eastern Cooperative Oncology Group
FACT-H&N: Functional Assessment of Cancer Therapy Head and Neck
FAS: Full Analysis Set
ICF: Informed Consent Form
ITT: Intent-to-Treat
GFR: Glomerular Filtration Rate
GTV: Gross Tumor Volume
IEC: Independent Ethics Committee
IMRT: Intensity Modulated Radiation Therapy
IRB: Institutional Review Board
JCCC: Jonsson Comprehensive Cancer Center
MRI: Magnetic Resonance Imaging
HIPAA: Health Insurance Portability and Accountability Act
MV: Megavoltage
OAR: Organ-at-Risk
ORC: Office of Regulatory Compliance
PET: Positron Emission Tomography
PI: Principal Investigator
PTV: Planning Target Volume
PULSAR: Personalized Ultrafractionated Stereotactic Adaptive Radiotherapy
SAE: Serious Adverse Event
SAS: Safety Analysis Set
SBRT: Stereotactic Body Radiotherapy
HNC: Head and Neck Cancer
SPIRIT: Standard Protocol Items: Recommendations for Interventional Trials
PFS: Progression-Free Survival
OS: Overall Survival
UCLA: University of California, Los Angeles
UW-QoL: University of Washington Quality of Life
VMAT: Volumetric Modulated Arc Therapy
WOCBP: Women of Child Bearing Potential
