# Supplementary material for: A phase II study of personalized ultrafractionated stereotactic adaptive radiotherapy for palliative head and neck cancer treatment (PULS-Pal): a single-arm clinical trial protocol
Source: BMC Cancer. 2024 Dec 21;24:1564. doi: 10.1186/s12885-024-13303-5 (PMC11662514; doi:10.1186/s12885-024-13303-5)
Supplement: Supplementary file 1 — Supplementary Material 1. [file 12885_2024_13303_MOESM1_ESM.docx]

**A phase II study of personalized ultrafractionated stereotactic adaptive radiotherapy for palliative head and neck cancer treatment (PULS-Pal): a single-arm clinical trial protocol.**

Supplemental Material

**Pages 2-11:** Informed Consent Form

**Pages 12-17:** University of Washington Quality of Life (UW-QOL v4.1) Questionnaire

**Pages 18-23:** Functional Assessment of Cancer Therapy Head and Neck (FACT-H&N) (Version 4) Questionnaire

**UNIVERSITY OF CALIFORNIA LOS ANGELES**

**CONSENT TO PARTICIPATE IN RESEARCH**

**Personalized ultrafractionated stereotactic adaptive radiotherapy for palliative head and neck cancer treatment (PULS-Pal)**

**A Study to See if a Radiation Treatment Planning Program and New Schedule Can Deliver a Higher Dose of Radiation with Better Tumor Control and the Same or Lesser Side Effects Than a Routine Dose and Schedule**

**INTRODUCTION**

P. Travis Courtney, MD, MAS, Robert Chin, MD, PhD, and associates from the Radiation Oncology Department at the University of California, Los Angeles, are conducting a research study. UCLA is the Sponsor of this study and is receiving funding from Varian Systems, Inc.

**KEY INFORMATION**

The following is a short summary of this study to help you decide whether or not to be a part of this study. More detailed information is listed later on in this form.

**WHY AM I BEING INVITED TO TAKE PART IN A RESEARCH STUDY?**

We invite you to take part in a research study because you have been diagnosed with new or recurrent, localized or metastatic head and neck cancer and are not eligible or decline standard of care treatment(s), including Stereotactic Body Radiation Therapy (SBRT.)

WHAT SHOULD I KNOW ABOUT A RESEARCH STUDY?

1. Someone will explain this research study to you.
2. Whether or not you take part is up to you.
3. You can choose not to take part.
4. You can agree to take part and later change your mind.
5. Your decision will not be held against you.
6. You can ask all the questions you want before you decide.
7. You can discuss this study with friends and family.
8. You can also discuss it with your healthcare doctor or request a second opinion.

WHY IS THIS RESEARCH BEING DONE?

This study aims to provide palliative tumor control of head and neck (HNC) cancer for people who can either not or decline to receive standard-of-care treatment.

In this study, we will evaluate if using HyperArc^TM^ and PULSAR (SBRT doses delivered “pulses” every 2 weeks) will deliver higher, possibly more effective doses, resulting in better tumor control with the same or fewer side effects than smaller routine doses. HyperArcTM is a radiation treatment planning system by Varian Systems, Inc.

You will be asked to complete a simulation scan so the treatment team can create a palliative treatment plan. You will start the study treatment about a week later, which includes five separate fractions (treatment doses) every two weeks. Once you complete the PULSAR radiation fractions, the treatment team will check in and ask you to complete study questionnaires. Participation in this study will last about 62 weeks, with 10 weeks of active radiation treatment and 52 weeks of follow-up. More detailed information about the study procedures can be found under ***“*WHAT WILL HAPPEN IF I TAKE PART IN THIS STUDY?*”***

WHAT KINDS OF RISKS OR DISCOMFORTS COULD I EXPECT?

Risks of Radiation Treatment to the Head and Neck:

Radiation to the head and neck includes risks and side effects. If you go on to receive radiation therapy based on the HyperArc^TM^ and PULSAR plan, you will receive a higher than routine dose. While we expect the dose to healthy structures using HyperArc^TM^ and PULSAR will be controlled so that side effects are the same or lower as a routine treatment, we cannot be certain. It is possible, therefore, that these side effects may be worse than if you receive a routine dose.

PULSAR side effects and risks include:

Very common – out of 100 people who receive PULSAR for head and neck cancer, 20 or more people may experience the following:

- Fatigue
- Mouth and gum sores
- Sore throat
- Stiffness and pain in the jaw
- Pain or swelling on or near the treatment site
- Skin changes, including redness, irritation, itchiness or dryness
- Temporary or permanent voice changes such as hoarseness

Common – out of 100 people who receive PULSAR for head and neck cancer, at least 5 but less than 20 people may experience the following:

- Dry mouth
- Difficulty and painful swallowing. In some instances, swallowing can be so difficult that it prevents the patient from eating, requiring the temporary placement of a “g-tube” into the stomach to deliver food
- Temporary or permanent change in or loss of taste and/or smell
- Temporary or permanent hair loss
- A type of swelling called lymphedema
- Temporary or permanent change in hearing

Uncommon – out of 100 people who receive PULSAR for head and neck cancer, at least 1 but less than 5 people may experience the following:

- Nausea or vomiting
- Bleeding, sometimes fatal
- Damage to the thyroid gland
- A tightening and/or abnormal opening between your air tract and digestive tract
- An abnormal opening between you mouth or digestive track and your skin

Rare – out of 100 people who receive PULSAR for head and neck cancer, less than 1 person may experience the following:

- A new cancer
- Damage to the spinal cord and/or nearby nerves

The risk of certain side effects depends on where your tumor is located in the head and neck. The risk of side effects may be higher if you have previously received radiation therapy to the head or neck. More detailed information about the risks of this study can be found under ***“*WHAT KINDS OF RISKS OR DISCOMFORTS COULD I EXPECT?  *(Detailed Description)”***

**ARE THERE ANY BENEFITS IF I PARTICIPATE?**

You may or may not benefit from the study. Everyone in the study will receive palliative care for their HNC. We hope that treatment planning with HyperArc^TM^ and the PULSAR schedule will allow the tumor to receive a higher dose while keeping the patient's side effects the same or less than routine care. The higher dose may be more effective in preventing tumor regrowth.

The research results may also benefit society. If planning with PULSAR is determined to be safe and effective, it may be used for future patients with rHNC or other cancers.

**WHAT OTHER CHOICES DO I HAVE IF I DON’T WANT TO PARTICIPATE?**

Participation in research is completely voluntary. You can decide to participate or not to participate. Your other choices may include:

- Receiving palliative treatment or care for your cancer without being in a study, including repeat conventional radiation
- Taking part in another study
- Receiving no treatment

**Disclosure Statement**

Your healthcare provider may be an investigator of this research protocol and, as an investigator, is interested in both your clinical welfare and in the conduct of this study. You are not under any obligation to participate in any research project offered by your clinician.

**HOW MANY PEOPLE WILL TAKE PART IN THIS STUDY?**

43 people will take part in this study at UCLA.

**WHAT WILL HAPPEN IF I TAKE PART IN THIS STUDY?**

If you volunteer to participate in this study and you are confirmed to be eligible to participate, the researcher will ask you to do the following:

Simulation and Planning:

- You will undergo a CT scan. You may be asked to receive an infusion of a contrast agent, delivered through a tube attached to a needle in your arm, before your CT scan. This process is routine and is called “Simulation.”
- You may need to repeat the CT simulation scan if your tumor changes in size during the radiation course.
- Over the next few days, your doctor will develop a radiation treatment plan for you.

Treatment:

About one week after your Simulation visit, you will return to the Department of Radiation Oncology to begin your treatment. You will receive five separate doses, or fractions, every two-week period, requiring five separate visits.

Follow-up Visits:

- You will also be asked to return to see your doctor at the Department of Radiation Oncology approximately one month, three months, six months, twelve months, eighteen months, and twenty-four months after you complete your treatment for routine check-ups and to see how you are doing. We will only collect information regarding your cancer and survival.
- At each of these follow-up visits, you will be asked to complete two questionnaires about how you are feeling before and during your treatment follow-up visits.
- In some instances, you and your doctor may conduct the follow-up visits by telephone/telemedicine. In some instances, you can complete the questionnaires over the phone, through an online system, or ask you to complete them by hand and return them to the study team.

**WHAT KINDS OF RISKS OR DISCOMFORTS COULD I EXPECT? (Detailed Description)**

**Known risks and discomforts:**

The possible risks and/or discomforts associated with the procedures described in this consent form include:

*Risks of Radiation Treatment to the Head and Neck:*

Radiation to the head and neck includes risks and side effects. If you go on to receive radiation therapy based on the PULSAR plan, you will receive a higher than routine dose. While we expect the dose to healthy structures using PULSAR will be controlled so that side effects are the same or lower as routine treatment, we cannot be certain. It is possible, therefore, that these side effects may be worse than if you receive a routine dose.

*Risks of CT and PET Scans*

The additional radiation exposure from the CT or PET/CT required by this study, and which are considered routine for your care, are within the acceptable limits for diagnostic studies involving ionizing radiation and are routinely used in medicine.

*Risks of Questionnaires:*

You will be asked to complete two questionnaires at most of your visits, as described above. You may find some of the questions asked in the questionnaires embarrassing or upsetting. Each questionnaire will take about 3 – 4 minutes to complete.

**Unknown risks and discomforts:**

The experimental treatments may have side effects that no one knows about yet. The researchers will let you know if they learn anything that might make you change your mind about participating in the study.

**CAN THE RESEARCHERS REMOVE ME FROM THIS STUDY?**

The researchers may end your participation in this study for a number of reasons, such as if your safety and welfare are at risk, if you do not follow instructions or if you miss scheduled visits. The researchers or the study sponsor might also decide to stop the study at any time.

If you decide to stop being in the study, or are removed from the study, or the study is stopped the researcher will ask you to complete an exit telephone interview.

**HOW WILL INFORMATION ABOUT ME AND MY PARTICIPATION BE KEPT CONFIDENTIAL?**

The researchers will do their best to make sure that your private information is kept confidential. Information about you will be handled as confidentially as possible, but participating in research may involve a loss of privacy and the potential for a breach in confidentiality. Study data will be physically and electronically secured. As with any use of electronic means to store data, there is a risk of breach of data security.

**Use of personal information that can identify you:**

Your data will be de-identified before being analyzed for research purposes. Any information that can link it to you will be removed.

**How information about you will be stored:**

Your data will be stored on a user-secured database at UCLA that is also firewall-protected. All data will be de-identified (anonymized) before being uploaded to another secure research database where it will be stored and downloaded for research purposes.

**People and agencies that will have access to your information:**

The research team, authorized UCLA personnel, the study sponsor, and regulatory agencies such as the Food and Drug Administration (FDA), may have access to study data and records to monitor the study. Research records provided to authorized, non-UCLA personnel will not contain identifiable information about you. Publications and/or presentations that result from this study will not identify you by name.

Employees of the University may have access to identifiable information as part of routine processing of your information, such as lab work or processing payment. However, University employees are bound by strict rules of confidentiality.

Because this study involves the treatment of a medical condition and/or medical procedures, a copy of this consent form will be placed in your medical record.  This will allow the doctors that are caring for you to obtain information about what medications and/or procedures you are receiving in the study and treat you appropriately.

**How long information from the study will be kept:**

Study data will be kept indefinitely.

**USE OF DATA AND SPECIMENS FOR FUTURE RESEARCH**

My data and/or specimens, including de-identified data and/or specimens, may be kept for use in future research.

**ARE THERE ANY COSTS FOR TAKING PART IN THIS STUDY?**

The study will pay for research-related items and/or services that are provided only because you are participating in the study. These research-related items and/or services are explained in other areas of this consent form.

You or your health plan may be responsible for paying for all the types of items listed below:

- Items and services that would have been provided to you even if you were not in the study
- Health care given during the study as part of your regular care
- Items or services needed to give you study drugs or devices
- Monitoring for side effects or other problems
- Deductibles or co-pays for these items and/or services

**WILL I BE PAID FOR MY PARTICIPATION?**

You will not be paid for your participation in this research study.

**RESEARCHER FINANCIAL INTERESTS IN THIS STUDY**

Dr. Cao, the investigator conducting this study, has a financial interest in Varian Systems, Inc., the company that manufactures and sells HyperArc^TM^ planning software being tested in this study.

**WHO CAN I CONTACT IF I HAVE QUESTIONS ABOUT THIS STUDY?**

**The Research Team:**

You may contact Travis Courtney, MD, MAS and/or Robert Chin, MD, PhD at 310-825-6577 with any questions or concerns about the research or your participation in this study. You can also call the UCLA Page Operator at (310) 825-6301 to reach the study clinicians 24 hours a day, 7 days week.

**UCLA Office of the Human Research Protection Program (OHRPP):**

If you have questions about your rights while taking part in this study, or you have concerns or suggestions and you want to talk to someone other than the researchers about the study, you may contact the UCLA OHRPP by phone: (310) 206-2040; by email: [participants@research.ucla.edu](mailto:participants@research.ucla.edu) or U.S. mail: UCLA OHRPP, Box 951406, Los Angeles, CA 90095-1406.

**Public Information about this Study:**

*ClinicalTrials.gov* is a website that provides information about federally and privately supported clinical trials. A description of this clinical trial will be available on [http://www.ClinicalTrials.gov](http://www.clinicaltrials.gov/), as required by U.S. Law. This website will not include information that can identify you. At most, the website will include a summary of the results. You can search this website at any time.

**WHAT HAPPENS IF I BELIEVE I AM INJURED BECAUSE I TOOK PART IN THIS STUDY?**

It is important that you promptly tell the researchers if you believe that you have been injured because of taking part in this study. You can tell the researcher in person or call him/her at the number(s) listed above.

If you are injured as a result of being in this study, UCLA will provide the necessary medical treatment. The costs of the treatment may be covered by the University of California or the study sponsor or billed to you or your insurer just like other medical costs, depending on a number of factors. The University and the study sponsor do not normally provide any other form of compensation for injury.  For more information about this, you may call the UCLA Office of the Human Research Protection Program at (310) 206-2040 or send an email to [participants@research.ucla.edu](mailto:participants@research.ucla.edu).

**WHAT ARE MY RIGHTS IF I TAKE PART IN THIS STUDY?**

Taking part in this study is your choice. You can choose whether or not you want to participate. Whatever decision you make, there will be no penalty to you and you will not lose any of your regular benefits.

- You have a right to have all of your questions answered before deciding whether to take part.
- Your decision will not affect the medical care you receive from UCLA.
- If you decide to take part, you can leave the study at any time.
- If you decide to stop being in this study you should notify the research team right away. The researchers may ask you to complete some procedures in order to protect your safety.
- If you decide not to take part, you can still get medical care from UCLA.

**HOW DO I INDICATE MY AGREEMENT TO PARTICIPATE?**

If you want to participate in this study you should sign and date below. You have been given a copy of this consent form and the Research Participant’s Bill of Rights to keep. You will be asked to sign a separate form authorizing access, use, creation, or disclosure of health information about you.

**SIGNATURE OF THE PARTICIPANT**

______________________________________

Name of Participant

______________________________________ ______________________

Signature of Participant Date

**SIGNATURE OF PERSON OBTAINING CONSENT**

______________________________________

Name of Person Obtaining Consent

______________________________________ ______________________

**University of Washington Quality of Life Questionnaire**

**(UW-QOL v4.1)**

This questionnaire asks about your health and quality of life **over the past seven days**. Please answer all of the questions by ticking one box for each question.

1. **Pain**. (Tick one box: 🗹 )

I have no pain. (100)

There is mild pain not needing medication. (75)

I have moderate pain - requires regular medication (e.g. paracetamol). (50)

I have severe pain controlled only by prescription medicine (e.g. morphine). (25)

I have severe pain, not controlled by medication. (0)

2. **Appearance**. (Tick one box: 🗹 )

There is no change in my appearance. (100)

The change in my appearance is minor. (75)

My appearance bothers me but I remain active. (50)

I feel significantly disfigured and limit my activities due to my appearance. (25)

I cannot be with people due to my appearance. (0)

3. **Activity**. (Tick one box: 🗹 )

I am as active as I have ever been. (100)

There are times when I can't keep up my old pace, but not often. (75)

I am often tired and have slowed down my activities although I still get out. (50)

I don't go out because I don't have the strength. (25)

I am usually in bed or chair and don't leave home. (0)

4. **Recreation**. (Tick one box: 🗹 )

There are no limitations to recreation at home or away from home. (100)

There are a few things I can't do but I still get out and enjoy life. (75)

There are many times when I wish I could get out more, but I'm not up to it. (50)

There are severe limitations to what I can do, mostly I stay at home and
 watch TV (25)

I can't do anything enjoyable. (0)

5. **Swallowing**. (Tick one box: 🗹 )

I can swallow as well as ever. (100)

I cannot swallow certain solid foods. (70)

I can only swallow liquid food. (30)

I cannot swallow because it "goes down the wrong way" and chokes me. (0)

6. **Chewing**. (Tick one box: 🗹 )

I can chew as well as ever. (100)

I can eat soft solids but cannot chew some foods. (50)

I cannot even chew soft solids. (0)

7. **Speech**. (Tick one box: 🗹 )

My speech is the same as always. (100)

I have difficulty saying some words but I can be understood over the phone. (70)

Only my family and friends can understand me. (30)

I cannot be understood. (0)

8. **Shoulder**. (Tick one box: 🗹 )

I have no problem with my shoulder. (100)

My shoulder is stiff but it has not affected my activity or strength. (70)

Pain or weakness in my shoulder has caused me to change my
 work / hobbies. (30)

I cannot work or do my hobbies due to problems with my shoulder. (0)

9. **Taste**. (Tick one box: 🗹 )

I can taste food normally. (100)

I can taste most foods normally. (70)

I can taste some foods. (30)

I cannot taste any foods. (0)

10. **Saliva**. (Tick one box: 🗹 )

I have too much saliva (30)

My saliva is of normal consistency (100)

I have less saliva than normal, but it is enough. (70)

I have too little saliva. (30)

I have no saliva. (0)

11. **Mood**. (Tick one box: 🗹 )

My mood is excellent and unaffected by my cancer. (100)

My mood is generally good and only occasionally affected by my cancer. (75)

I am neither in a good mood nor depressed about my cancer. (50)

I am somewhat depressed about my cancer. (25)

I am extremely depressed about my cancer. (0)

12. **Anxiety**. (Tick one box: 🗹 )

I am not anxious about my cancer. (100)

I am a little anxious about my cancer. (70)

I am anxious about my cancer. (30)

I am very anxious about my cancer. (0)

Which issues have been the most important to you during the past 7 days?

Tick 🗹 **up to 3 boxes.**

Pain Swallowing Taste

Appearance Chewing Saliva

Activity Speech Mood

Recreation Shoulder Anxiety

13. **Intimacy**. (Tick one box: 🗹 )

I have no problem with intimacy as a result of my cancer (100)

I have problems with intimacy but it does not bother me very much (70)

I have problems with my intimacy and this causes me some concern (30)

I have major problems with intimacy and this causes me considerable concern (0)

14. **Fear of cancer recurrence**. (Tick one box: 🗹 )

I have no fear of recurrence (100)

I have a little fear, with occasional thoughts but they don’t really bother me (75)

I am sometimes having fearful thoughts but I can usually manage these (50)

I get a lot of fears of recurrence and these can really preoccupy my thoughts (25)

I am fearful all the time that my cancer might return and I struggle with this (0)

Which of these issues have been important to you during the past 7 days? Tick 🗹 **up to 2 boxes.**

Intimacy  Fear of Recurrence 

**GENERAL QUESTIONS**

**Compared to the month before you developed cancer**, how would you rate your health-related quality of life? (Tick one box: 🗹 )

Much better (100)

Somewhat better (75)

About the same (50)

Somewhat worse (25)

Much worse (0)

In general, would you say your **health-related quality of life** during the past 7 days has been: (Tick one box: 🗹 )

Outstanding (100)

Very good (80)

Good (60)

Fair (40)

Poor (20)

Very poor (0)

Overall quality of life includes not only physical and mental health, but also many other factors, such as family, friends, spirituality, or personal leisure activities that are important to your enjoyment of life. Considering everything in your life that contributes to your personal well-being, rate your **overall quality of life** during the past 7 days. (Tick one box: 🗹 )

Outstanding (100)

Very good (80)

Good (60)

Fair (40)

Poor (20)

Very poor (0)

Please describe any other issues (medical or nonmedical) that are important to your quality of life and have not been adequately addressed by our questions (you may attach additional sheets if needed).

**Functional Assessment of Cancer Therapy Head and Neck**

**(FACT-H&N) (Version 4)**

Below is a list of statements that other people with your illness have said are important**. Please circle or mark one number per line to indicate your response as it applies to the past 7 days.**

|  | **PHYSICAL WELL-BEING** | **Not at all** | **A little bit** | **Some-what** | **Quitea bit** | **Very much** |
| --- | --- | --- | --- | --- | --- | --- |
|  |  |  |  |  |  |  |
| GP1 | I have a lack of energy | 0 | 1 | 2 | 3 | 4 |
| GP2 | I have nausea | 0 | 1 | 2 | 3 | 4 |
| GP3 | Because of my physical condition, I have trouble meeting the needs of my family | 0 | 1 | 2 | 3 | 4 |
| GP4 | I have pain | 0 | 1 | 2 | 3 | 4 |
| GP5 | I am bothered by side effects of treatment | 0 | 1 | 2 | 3 | 4 |
| GP6 | I feel ill | 0 | 1 | 2 | 3 | 4 |
| GP7 | I am forced to spend time in bed | 0 | 1 | 2 | 3 | 4 |
|  | | | | | | |

|  | **SOCIAL/FAMILY WELL-BEING** | **Not at all** | **A little bit** | **Some-what** | **Quitea bit** | **Very much** |
| --- | --- | --- | --- | --- | --- | --- |
|  |  |  |  |  |  |  |
| GS1 | I feel close to my friends | 0 | 1 | 2 | 3 | 4 |
| GS2 | I get emotional support from my family | 0 | 1 | 2 | 3 | 4 |
| GS3 | I get support from my friends | 0 | 1 | 2 | 3 | 4 |
| GS4 | My family has accepted my illness | 0 | 1 | 2 | 3 | 4 |
| GS5 | I am satisfied with family communication about my illness | 0 | 1 | 2 | 3 | 4 |
| GS6 | I feel close to my partner (or the person who is my main support) | 0 | 1 | 2 | 3 | 4 |
| Q1 | *Regardless of your current level of sexual activity, please answer the following question. If you prefer not to answer it, please mark this box and go to the next section.* |  |  |  |  |  |
| GS7 | I am satisfied with my sex life | 0 | 1 | 2 | 3 | 4 |

**Please circle or mark one number per line to indicate your response as it applies to the past 7 days.**

|  | **EMOTIONAL WELL-BEING** | **Not at all** | **A little bit** | **Some-what** | **Quitea bit** | **Very much** |
| --- | --- | --- | --- | --- | --- | --- |
|  |  |  |  |  |  |  |
| GE1 | I feel sad | 0 | 1 | 2 | 3 | 4 |
| GE2 | I am satisfied with how I am coping with my illness | 0 | 1 | 2 | 3 | 4 |
| GE3 | I am losing hope in the fight against my illness | 0 | 1 | 2 | 3 | 4 |
| GE4 | I feel nervous | 0 | 1 | 2 | 3 | 4 |
| GE5 | I worry about dying | 0 | 1 | 2 | 3 | 4 |
| GE6 | I worry that my condition will get worse | 0 | 1 | 2 | 3 | 4 |

|  | **FUNCTIONAL WELL-BEING** | **Not at all** | **A little bit** | **Some-what** | **Quitea bit** | **Very much** |
| --- | --- | --- | --- | --- | --- | --- |
|  |  |  |  |  |  |  |
| GF1 | I am able to work (include work at home) | 0 | 1 | 2 | 3 | 4 |
| GF2 | My work (include work at home) is fulfilling | 0 | 1 | 2 | 3 | 4 |
| GF3 | I am able to enjoy life | 0 | 1 | 2 | 3 | 4 |
| GF4 | I have accepted my illness | 0 | 1 | 2 | 3 | 4 |
| GF5 | I am sleeping well | 0 | 1 | 2 | 3 | 4 |
| GF6 | I am enjoying the things I usually do for fun | 0 | 1 | 2 | 3 | 4 |
| GF7 | I am content with the quality of my life right now | 0 | 1 | 2 | 3 | 4 |

**Please circle or mark one number per line to indicate your response as it applies to the past 7 days.**

|  | **ADDITIONAL CONCERNS** | **Not at all** | **A little bit** | **Some-what** | **Quite a bit** | **Very much** |
| --- | --- | --- | --- | --- | --- | --- |
|  |  |  |  |  |  |  |
|  |  |  |  |  |  |  |
|  |  |  |  |  |  |  |
| H&N1 | I am able to eat the foods that I like | 0 | 1 | 2 | 3 | 4 |
| H&N2 | My mouth is dry | 0 | 1 | 2 | 3 | 4 |
| H&N3 | I have trouble breathing | 0 | 1 | 2 | 3 | 4 |
| H&N4 | My voice has its usual quality and strength | 0 | 1 | 2 | 3 | 4 |
| H&N5 | I am able to eat as much food as I want | 0 | 1 | 2 | 3 | 4 |
| H&N6 | I am unhappy with how my face and neck look | 0 | 1 | 2 | 3 | 4 |
| H&N7 | I can swallow naturally and easily | 0 | 1 | 2 | 3 | 4 |
| H&N8 | I smoke cigarettes or other tobacco products | 0 | 1 | 2 | 3 | 4 |
| H&N9 | I drink alcohol (e.g. beer, wine, etc.) | 0 | 1 | 2 | 3 | 4 |
| H&N 10 | I am able to communicate with others | 0 | 1 | 2 | 3 | 4 |
| H&N 11 | I can eat solid foods | 0 | 1 | 2 | 3 | 4 |
| H&N 12 | I have pain in my mouth, throat or neck | 0 | 1 | 2 | 3 | 4 |
